# Molecular Characteristics of m6A Regulators and Tumor Microenvironment Infiltration in Soft Tissue Sarcoma: A Gene-Based Study

**DOI:** 10.3389/fbioe.2022.846812

**Published:** 2022-04-19

**Authors:** Kang-Wen Xiao, Zhi-Qiang Yang, Xin Yan, Zhi-Bo Liu, Min Yang, Liang-Yu Guo, Lin Cai

**Affiliations:** ^1^ Department of Orthopedics, Zhongnan Hospital of Wuhan University, Wuhan, China; ^2^ Department of Urology, Zhongnan Hospital of Wuhan University, Wuhan, China

**Keywords:** m6A, soft tissue sarcoma, immunotherapy, tumor microenvironment, survival

## Abstract

**Background:** N6-methyladenosine (m6A) methylation played a key role in tumor growth. However, the relationship between m6A and soft tissue sarcoma (STS) was still unclear.

**Methods:** The characterization and patterns of m6A modification in STS (TCGA-SARC and GSE17674) were analyzed comprehensively through bioinformatics and real-time polymerase chain reaction (RT-PCR). The effects of different m6A modification patterns on prognosis and immune infiltration of STS were further explored. Differentially expressed gene (DEG) analysis was performed. Moreover, an m6Ascore was constructed by principal component analysis (PCA). In addition, two immunotherapy datasets (IMvigor210 and GSE78220) and a sarcoma dataset (GSE17618) were used to evaluate the m6Ascore.

**Results:** Huge differences were found in somatic mutation, CNV, and expression of 25 m6A regulators in STS. Two modification patterns (A and B) in STS were further identified and the m6A cluster A showed a better clinical outcome with a lower immune/stromal score compared with the m6A cluster B (*p* < 0.050).In addition to , most STS samples from m6A cluster A showed a high m6Ascore, which was related to mismatch repair and a better prognosis of STS (*p* < 0.001). In contrast, the m6A cluster B, characterized by a low m6Ascore, was related to the MYC signaling pathway, which led to a poor prognosis of STS. A high m6Ascore also contributed to a better outcome of PD-1/PD-L1 blockade immunotherapy.

**Conclusion:** The modification patterns of 25 m6A regulators in the STS microenvironment were explored comprehensively. The novel m6Ascore effectively predicted the characteristics of the tumor microenvironment (TME) and outcome in STS and provided novel insights for future immunotherapy.

## Introduction

Since m6A was first detected in poly (A) RNA in 1974, this RNA modification has been found to regulate huge numbers of biological processes in many fields (Alarcón et al., 2015). Currently, m6A regulators could be divided into three types: adenosine methyltransferases (writers), demethylases (erasers), and binding proteins (readers) (Cao et al., 2016). Dysfunction of these regulators would contribute to incomplete m6A modification, which further regulated tumor development and progression (Delaunay and Frye, 2019). For instance, increased expression of ALKBH5 caused by hypoxia could accelerate mammosphere growth in cancer stem cells (Zhang et al., 2016). Furthermore, high expression of ALKBH5 also contributed to the inhibition of the progression of bladder cancer (Yu et al., 2021). A recent study demonstrated that overexpression of YTHDF1 could promote the growth of hepatocellular carcinoma through autophagy (Li et al., 2021a). Moreover, METTL3 was also found to accelerate tumorigenesis in glioblastoma cells (Cui et al., 2017). Although the role of m6A regulators in other tumors was widely studied recently, the relationship between m6A and STS remained unclear. Hence, it was of great importance to evaluate the role of m6A in STS.

Being rare mesenchymal malignancies with heterogeneity, STSs have been paid more attention in the last few decades (Stiller et al., 2013). The latest research calculated that nearly 13,130 new STS cases and 5,350 deaths would be detected in America (Siegel et al., 2020). However, the current treatment of STS is still surgery combined with radiotherapy, which led to distant metastasis in 25% of STS patients, and the metastasis rate rose to about 50% in high-grade STS (Brennan, 2005). Therefore, it was important to explore novel strategies against STS.

Immunotherapy, mainly consisting of PD-1/L1 and CTLA-4, has emerged as a promising treatment for cancer. However, the responsiveness to those immune checkpoint blockade (ICB) was low and few tumors were reported to effectively respond to ICB, which disappointed clinicians and patients (Topalian et al., 2012). The TME has been reported to be correlated with many tumor activities including tumor angiogenesis and growth (Hanahan and Coussens, 2012). As a key part of the TME, several immune cells have been observed to affect the progression and the response to cancer immunotherapy. Recent research revealed that excluded T cells could inhibit the tumor response to ICB (Mariathasan et al., 2018). Moreover, a recent study reported that decreased YTHDF1 expression was found to enhance the antitumor ability of CD8 (+) T cells in the mouse model (Wilkerson and Hayes, 2010). Although many components of the TME have been reported to be associated with tumor progression and immunotherapeutic effects, these studies were usually based on individual immune components and were not related to the m6A modification. Therefore, integrative analysis of m6A modification in the STS microenvironment and exploring effective markers to predict the therapeutic effect of ICB were urgently needed.

In this study, clinical and transcriptome data of STS from TCGA (The Cancer Genome Atlas) and GEO (Gene Expression Omnibus) databases were collected. Genetic variation and expression of m6A regulators in STS were further analyzed. The Search Tool for Recurring Instances of Neighboring Genes (STRING) database was used to detect connections among m6A regulators (Han et al., 2019). Then, two different m6A modification patterns were identified using consensus cluster analysis (Wilkerson and Hayes, 2010), a method that has been widely used in bioinformatics. Moreover, significantly different prognoses, immune infiltration, and pathways of STS were detected between these two m6A modification patterns. In addition, the m6Ascore accurately evaluated the prognosis and immunotherapy response of the tumor, which brought novel insights into the immunotherapy of STS.

## Methods

### Sample and Data Collection

TCGA-SARC with 265 STS samples and the corresponding clinical information, somatic mutation, and CNV were collected from UCSC-XENA (http://xena.ucsc.edu/). Here, we chose somatic mutation data to explore the somatic mutation of m6A regulators in STS, while CNV data were to explore the difference in CNV of m6A regulators in STS. Transcriptome data were used to explore the expression of m6A regulators between STS and adipose tissue and for further bioinformatics analysis. GSE17674 with 62 samples (Hugo et al., 2016) and GSE17618 (Savola et al., 2011) including 44 STS samples were collected from the GEO database (https://www.ncbi.nlm.nih.gov/geo/). The human genome annotation GTF file was collected from the gencode platform (https://www.gencodegenes.org/). GPL570 was used for GSE17618 and GSE17674. Robust multi-array average (RMA) normalization was performed in GSE17674 and GSE17618, while transcripts per kilobase million (TPM) normalization was performed in TCGA-SARC. Basic information on these three datasets were shown in Supplementary Table S1. Three pairs of STS samples and the adjacent normal tissue were collected from Zhongnan Hospital of Wuhan University. This study was also approved by the institutional ethics board of Zhongnan Hospital of Wuhan University.

Immunotherapy datasets IMvigor210 (anti-PD-L1) including 298 samples with complete clinical information and GSE78220 (anti-PD-1) (Hugo et al., 2016) including 27 samples with complete clinical information were collected from a previous study (Mariathasan et al., 2018) and GEO database, respectively. IMvigor210 was normalized by the trimmed mean of M-values, and GSE78220 was normalized by FPKM (Fragments Per Kilobase Million). A total of 25 m6A regulators were selected based on recent studies (Zhang et al., 2020; Chen et al., 2021). The flowchart of this study is displayed in Supplementary Figure S1.

### Cell Culture

Human skeletal muscle cell line (HSMC) and sarcoma cell line (A673) were collected from the American Type Culture Collection. A673 was cultured in RPMI 1640 medium (Hyclone) with 10% fetal bovine serum (Gibco) and 1% antibiotics (100 U/ml penicillin and 100 μg/ml streptomycin). HSMC was cultured in DMEM (Hyclone) medium with 10% fetal bovine serum and 1% antibiotics (100 U/ml penicillin and 100 μg/ml streptomycin). The cells were maintained in an incubator set to 37°C with 5% CO_2_ and passaged regularly.

### Real-Time Polymerase Chain Reaction

The total RNA of cell lines and tissue was extracted by the Trizol method (Invitrogen), and then, the RNA was reverse transcribed by using a reverse transcription kit (Roche) to obtain cDNA; the experimental operation was carried out according to the instructions of Trizol and the reverse transcription kit. RT-PCR was performed according to the instructions. The primer sequence of each m6A regulator is shown in Supplementary Table S2.

### Identification of Different N6-Methyladenosine Modification Patterns in Soft Tissue Sarcoma Through Consensus Cluster Analysis

The 25 m6A regulators included 15 readers (IGF2BP1, IGF2BP2, IGF2BP3, YTHDF1, YTHDF2, YTHDF3, YTHDC1, YTHDC2, FMR1, HNRNPA2B1, HNRNPC, RBMX, LRPPRC, ELAVL1, and EIF3A), eight writers (METTL3, METTL14, WTAP, VIRMA, RBM15, RBM15B, ZC3H13, andCBLL1) and two erasers (FTO and ALKBH5). Different m6A modification patterns in STS were determined by consensus cluster analysis in R software according to the expression of these m6A in TCGA-SARC. Furthermore, the aforementioned process was repeated 1,000 times to obtain a stable clustering effect by using the ConsensusClusterPlus R package (Wilkerson and Hayes, 2010).

### Differentially Expressed Gene Analysis, Protein–Protein Interaction Analysis, and Connectivity Map Analysis

Gene signatures of different m6A modification patterns were identified based on DEG analysis using the limma package (Ritchie et al., 2015) in R software. DEGs were also analyzed between normal samples and STSs in GSE17674. The Benjamini–Hochberg method (Storey, 2002) was used here to adjust multiple hypotheses. Adjust *p* < 0.050 and log^FC^ > 1 or log^FC^ < −1 were considered significant. The Connectivity Map used a genome-wide transcriptome system to comprehensively describe the biological status of the disease, physiology, and drug induction and further linked genes, drugs, and pathways (Lamb et al., 2006). The DEGs were further uploaded to the cMap database for drug prediction. *p* < 0.05 indicated statistical significance. In addition, 25 m6A regulators were used for PPI analysis and further visualized by Cytoscape (Shannon et al., 2003). The confidence level was 0.4.

### Characteristics of the Soft Tissue Sarcoma Microenvironment Based on Different N6-Methyladenosine Modification Patterns

CIBERSORT algorithm was used to evaluate the immune infiltration of TCGA-SARC (Chen et al., 2018). The permutations of the signature matrix were 1,000. The immune and stromal scores of STSs were evaluated by the ESTIMATE package (Yoshihara et al., 2013).

### Functional Enrichment Analysis

To further explore the differences in enrichment pathways among different m6A modifications, all genes from TCGA-SARC were used for gene set enrichment analysis (GSEA) based on different m6A modification patterns (A and B). Moreover, the clusterProfiler package (Yu et al., 2012) was used to screen significant pathways using Gene Ontology (GO) and Kyoto Encyclopedia of Genes and Genomes (KEGG) based on 25 m6A regulators and m6A modification-related DEGs, respectively. False discovery rate <0.05 and *p* < 0.05 were considered significant.

### Construction of the m6A Score

In order to quantify the modification patterns of m6A in STS, we constructed an m6A score learning from a previous study (Sotiriou et al., 2006). The specific procedures were as follows: first, the consensus cluster analysis was used to divide patients into several clusters according to DEGs between different m6A modification patterns; second, the prognostic DEGs were screened based on univariate Cox regression analysis; and finally, after z-score normalization, principal component analysis (PCA) was used to construct m6A score based on prognostic DEGs using principal component 1 as the signature score. The formula of the m6A score is shown as follows:

$$m6Ascore\;=\;\sum pc1_{m}-\sum pc1_{n},$$

where m represents the expression of prognostic DEGs with hazard ratio (HR) < 1, while n represents the expression of prognostic DEGs with HR > 1.

### Statistical Analysis

Statistical Product and Service Solutions software (SPSS 22.0) and R 3.6.2 were used for data analysis. The Maftool package (Mayakonda et al., 2018) was used to display the mutation landscape in TCGA-SARC, while the RCircos package (Krzywinski et al., 2009) was used to show the variation of 25 m6A regulators on human chromosomes. Pearson correlation analysis was performed using the corrplot package (https://cran.r-project.org/web/packages/corrplot/index.html) to assess the relationship among different m6A regulators and different immune cells, respectively. Cox regression analysis (Harrell et al., 1996) was performed along with Kaplan–Meier curve analysis to identify the prognostic m6A regulators and DEGs, respectively. For the survival analysis, a survival package was used and a cut-off point was set using the survminer package (Ranstam and Cook, 2017). Furthermore, different datasets were separately divided into different groups based on low and high m6A score, and prognostic differences were explored. All heatmaps were shown using the Pheatmap package (Wang et al., 2014) in R software. The survival rate was compared by the logrank test. Meanwhile, the receiver operating characteristic (ROC) curve for predicting the prognosis of TCGA-SARC and GSE17618 was performed by using the timeROC package (Blanche et al., 2013). PCA was performed using the FactoMineR package (Lê et al., 2008). The Kruskal–Wallis test was performed to compare differences between groups. All significance levels were *p* < 0.05.

## Results

### Landscape of N6-Methyladenosine Variation in Soft Tissue Sarcoma

**FIGURE 1:**
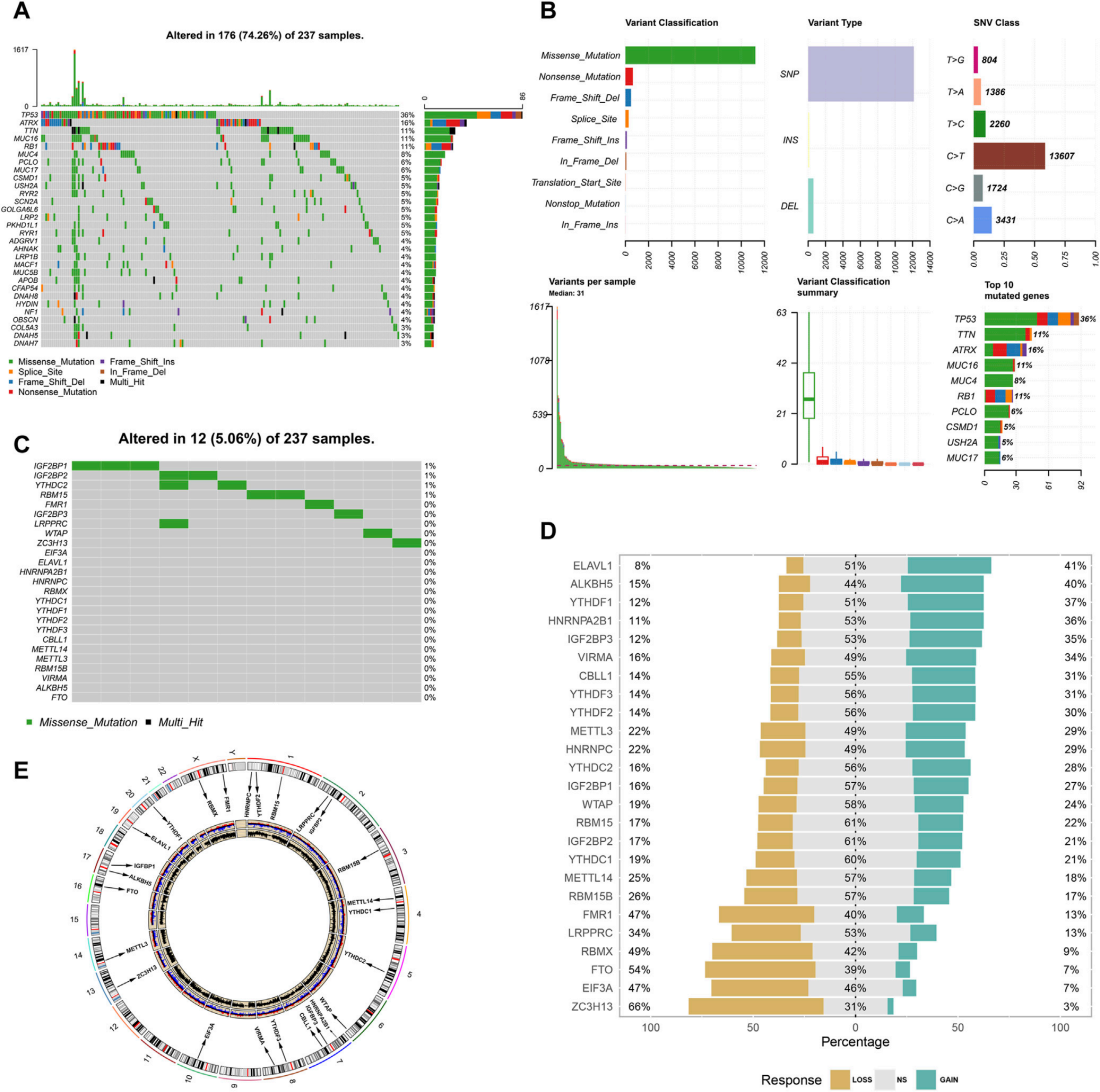
Landscape of somatic mutations and CNV of m6A regulators in STS. **(A)** Summary of somatic mutations in STS; **(B)** variant classifications of mutations in STS; **(C)** summary of somatic mutations of m6A regulators in STS; **(D)** CNV of 25 m6A regulators in STS; and **(E)** locations of different m6A regulators in human chromosomes.

**FIGURE 2:**
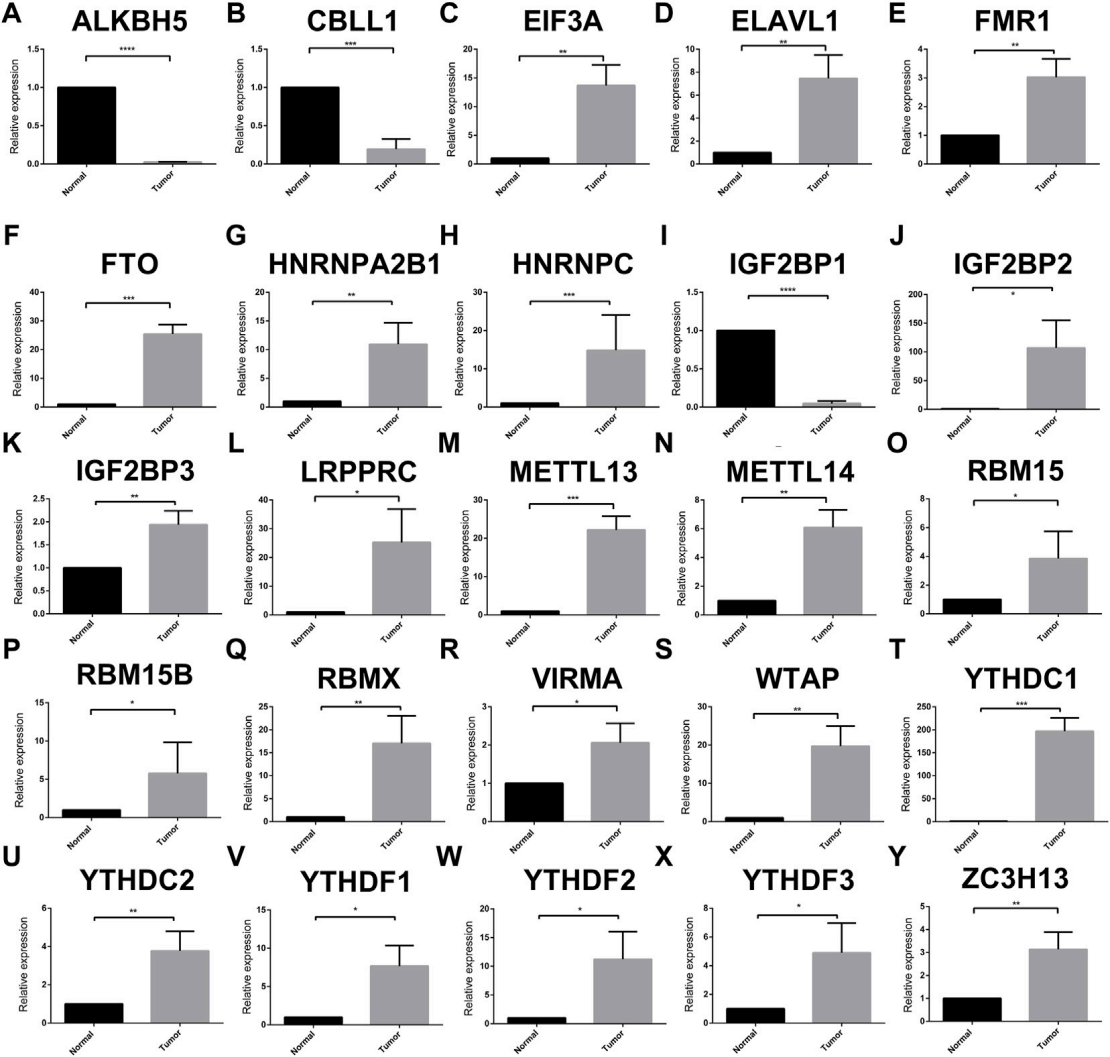
Expression of 25 m6A regulators between STS and normal adjacent tissue. **(A–Y)** Expression of different m6A regulators between normal adjacent tissue and STS samples. *p* < 0.05*, *p* < 0.01**, *p* < 0.001***, and *p* = 0****.

**FIGURE 3:**
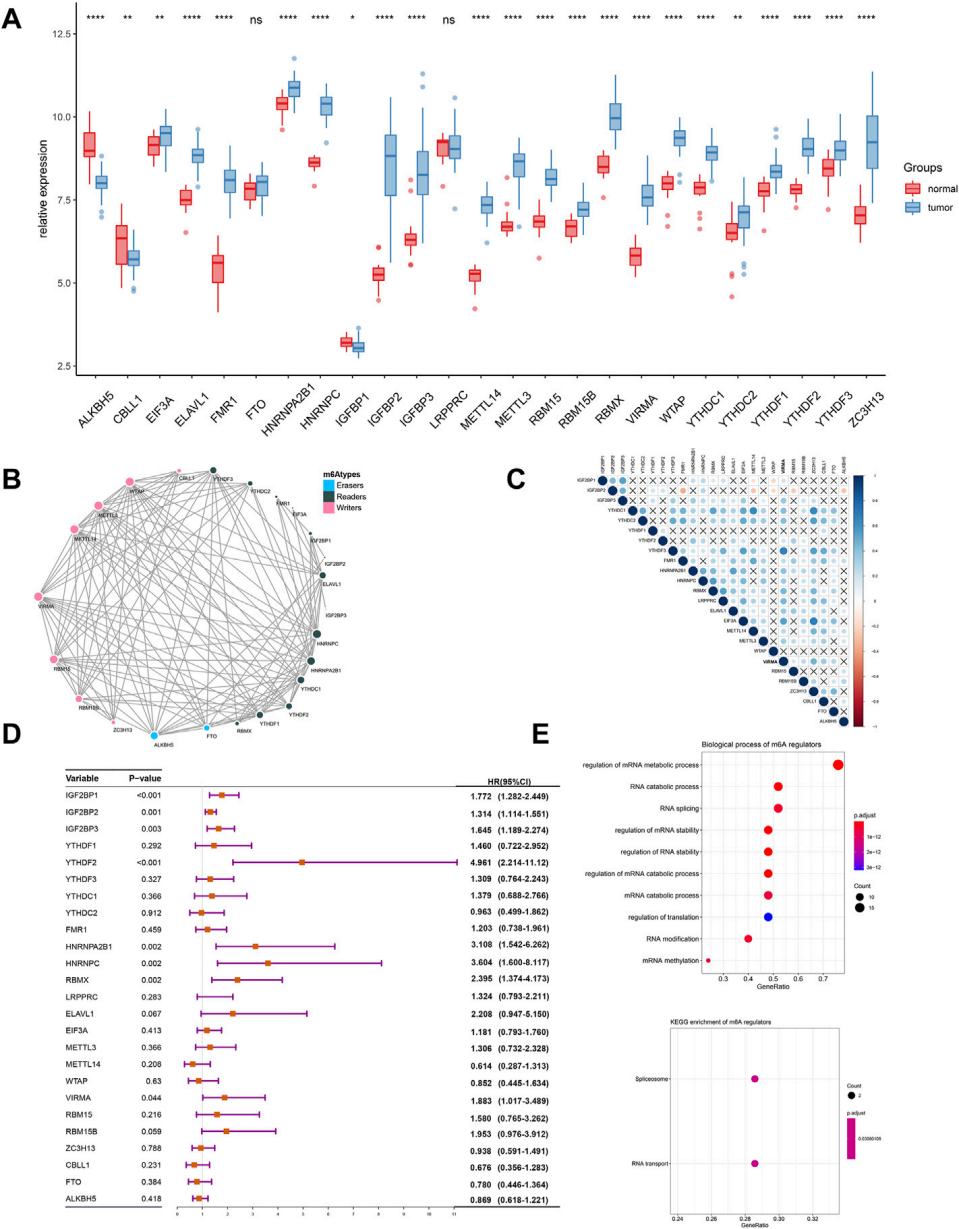
Expression, interactions, prognosis, and functional annotations of 25 m6A regulators in STS. **(A)** Expression of different m6A regulators between normal samples and STS samples using the GSE17674 dataset; **(B)** PPI analysis of 25 m6A regulators; and **(C)** correlation plot among 25 regulators using Pearson correlation analysis. *p* <0.010 indicated statistical significance. **(D)** Univariate Cox regression analysis for 25 m6A regulators in STS samples; **(E)** functional annotations for 25 m6A regulators. *p* <0.05*, *p* <0.01**, *p* <0.001***, and *p* = 0****; ns, no significance.

Somatic mutations in TCGA-SARC are shown in Figure 1A. Of the 237 samples, 176 were detected to have somatic mutations, accounting for 76.3% of the total. Furthermore, the mutation frequency of TP53, ATRX, and TTN was 36, 16, and 11%, respectively. The summary of variant classification and type is also shown in Figure 1B. The major variant classification, type, and single-nucleotide variant type were missense mutation, single-nucleotide polymorphism, and C-T transition, respectively. Considering the widespread somatic variation that existed in STS, the somatic mutations of m6A regulators are also shown in Figure 1C. Among 237 samples, only 12 samples had m6A mutations with low mutation frequency. The CNV alteration of 25 m6A regulators is also shown in Figure 1D. CNV generally occurred in every m6A regulator. Among them, ZC3H13 (66%), EIF3A (47%), FTO (54%), RBMX (49%), and FMR1 (47%) were found to have higher frequency of CNV gain. ELAVL1 (41%), ALKBH5 (40%), YTHDF1 (37%), HNRNPA2B1 (36%), and IGF2BP3 (35%) had higher frequency of CNV loss. Figure 1E also displays the location of different m6A regulators. We also explored the expression of these regulators at the cellular and tissue levels (Figure 2 and Supplementary Figure S2). The result turned out that a wide difference in m6A expression existed in STS. Compared with normal samples, ALKBH5, CBLL1, and IGFBP1 showed lower expression while most of the m6A regulators showed high expression. Considering huge differences in expression among the m6A regulators, we further compared the difference in m6A expression between normal samples and STSs in GSE17674, and the results were consistent with the abovementioned trend (Figure 3A). PPI was used to show interactions between m6A regulators, and the result isshown in Figure 3B. These m6A regulators were well connected to each other. The correlation plot of each m6A regulator is also displayed in Figure 3C. Most of the m6A regulators were correlated with each other, which was consistent with the result of PPI. In addition, univariate Cox analysis indicated that IGF2BP1 (*p* < 0.001), IGF2BP2 (*p* = 0.001), IGF2BP3 (*p* = 0.003), YTHDF2 (*p* < 0.001), HNRNPA2B1 (*p* = 0.002), HNRNPC (*p* = 0.002), RBMX (*p* = 0.002), and VIRMA (*p* = 0.044) were significantly correlated with the prognosis of STS. The abovementioned result is also shown in Figure 3D.In addition, , GO and KEGG pathways based on 25 m6A regulators were analyzed, and the results are shown in Figure 3E. These genes were enriched in RNA modification-associated pathways. Since widespread variation and expression of m6A regulators existed in STS, m6A regulators might play an important role in the progression and prognosis of STS.

### Identification of Two N6-Methyladenosine Modification Patterns in Soft Tissue Sarcoma

**FIGURE 4:**
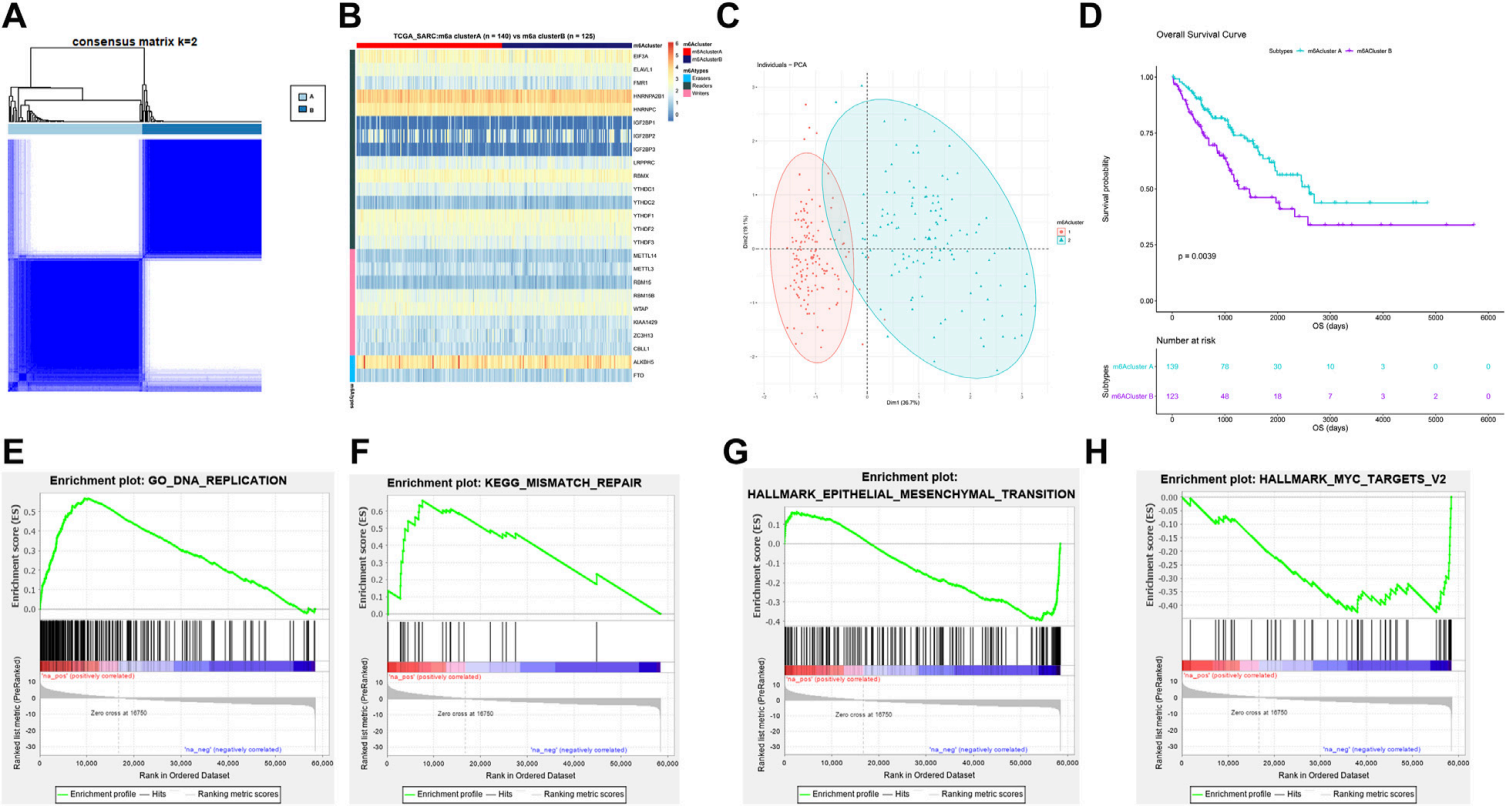
Identification of two m6A modification patterns in STS. **(A)** The result of consensus clustering analysis in STS; **(B)** heatmap of expression of 25 m6A regulators in m6A clusters A and B; **(C)** the result of PCA, indicating two distinct clusters were identified; **(D)** survival plot of two clusters in TCGA-SARC (*p* = 0.0039); **(E)** enriched pathways in m6A cluster A: DNA replication; **(F)** enriched pathways in m6A cluster A: mismatch repair; **(G)** enriched pathways in m6A cluster B: epithelial–mesenchymal transition; and **(H)** enriched pathways in m6A cluster B: MYC target.

To further analyze the effect of m6A modification on STS, we performed a consensus cluster analysis in TCGA-SARC. Two m6A modification patterns (A and B) were identified, and the result of clustering is shown in Figure 4A. The corresponding cumulative distribution function plot and delta area plot for clustering are also shown in Supplementary Figures S3A, B. m6A cluster A had 140 STS samples, while m6A cluster B had 125 samples. The heatmap of m6A expression among different modification patterns is also shown in Figure 4B. Among them, FMR1 (*p* < 0.001), HNRNPC (*p* = 0.001), IGF2BP1 (*p* < 0.001), IGF2BP2 (*p* < 0.001), IGF2BP3 (*p* < 0.001), YTHDC1 (*p* < 0.001), YTHDC2 (*p* < 0.001), YTHDF1 (*p* = 0.006), YTHDF1 (*p* = 0.013), METTL14 (*p* < 0.001), METTL3 (*p* = 0.001), RBM15 (*p* < 0.001), RBM15B (*p* = 0.010), WTAP (*p* = 0.010), and ALKBH5 (*p* = 0.010) were significantly differentially expressed in m6A cluster A and B. As in Figure 4C, the result of PCA also indicated that the consensus cluster well differentiated m6A cluster A and m6A cluster B. The result of survival analysis based on m6A clusters A and B is subsequently shown in Figure 4D. m6A cluster A had a significantly improved prognosis of STS than m6A cluster B (*p* = 0.004). The Cox regression analysis also indicated that different m6A clusters were correlated with the prognosis of STS (*p* = 0.004). The 5-year survival rate of m6A cluster A (63.5%) was significantly better than that of m6A cluster B (46.1%) with *p* = 0.038. The GSEA was also performed, and the result is shown in Figures 4E–H. Pathways associated with better prognosis were significantly enriched in m6A cluster A including DNA replication [false discovery rate (FDR) = 0; enrichment score (ES) = 0.721] and mismatch repair (FDR = 0; ES = 0.662). In contrast, pathways including epithelial–mesenchymal transition (EMT, *p* = 0, FDR = 0.001, ES = −0.390) and MYC signaling pathway (*p* = 0, FDR = 0.003, ES = −0.360) were enriched in m6A cluster B, which often led to poorer outcome of STS. Full lists of enriched pathways are shown in Supplementary Tables S3, S4. The aforementioned analysis revealed that different m6A modifications were associated with different pathways, which further affected the prognosis of STS.

### Characteristics of N6-Methyladenosine Modification Patterns in the Soft Tissue Sarcoma Microenvironment

**FIGURE 5:**
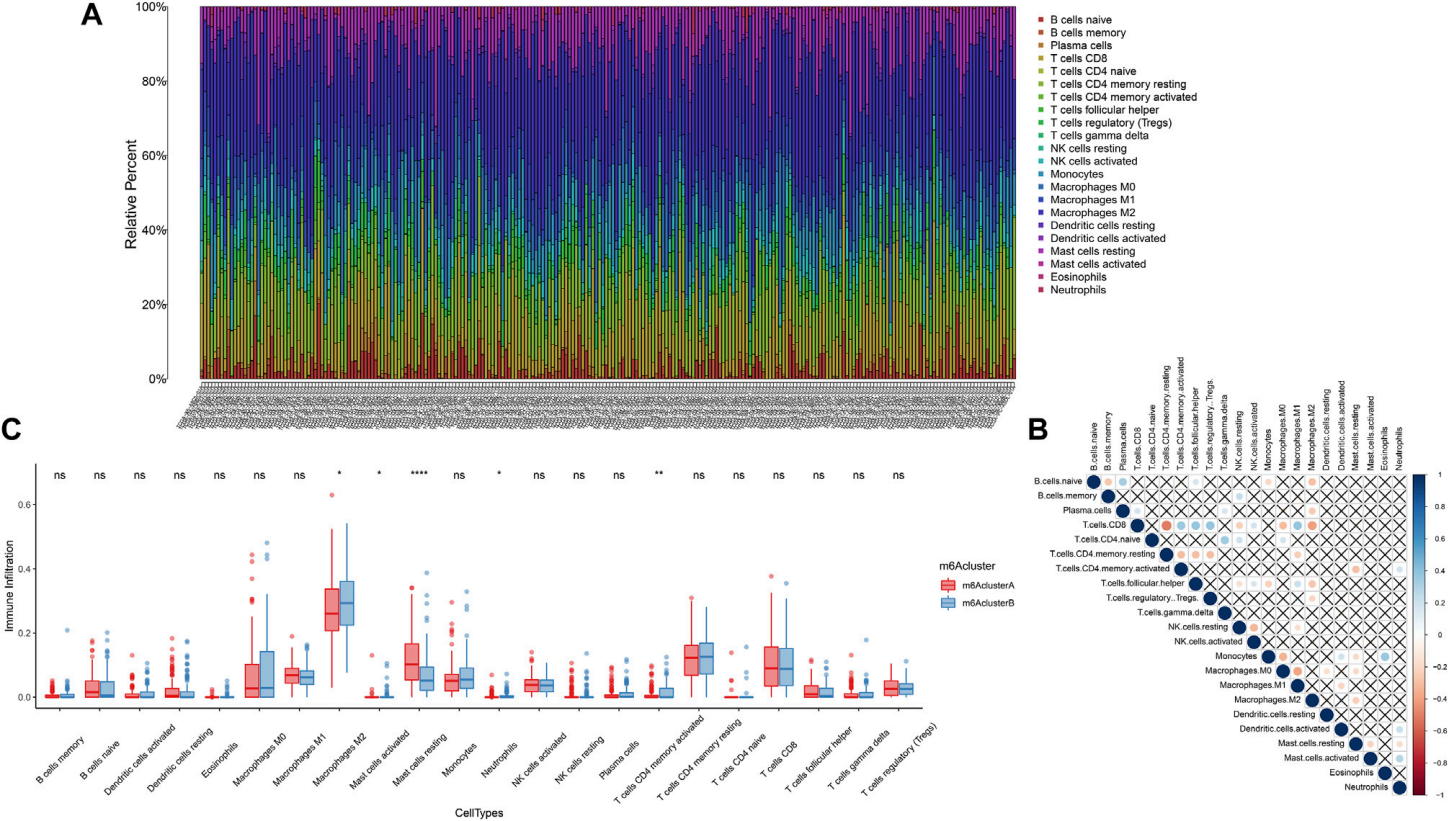
Effects of different m6A modification patterns on the immune infiltration of STS. **(A)** Immune infiltration of each sample in TCGA-SARC. **(B)** Correlation plot of each immune cell in TCGA-SARC. **(C)** Comparison of immune cell infiltration among m6A clusters A and B.

**FIGURE 6:**
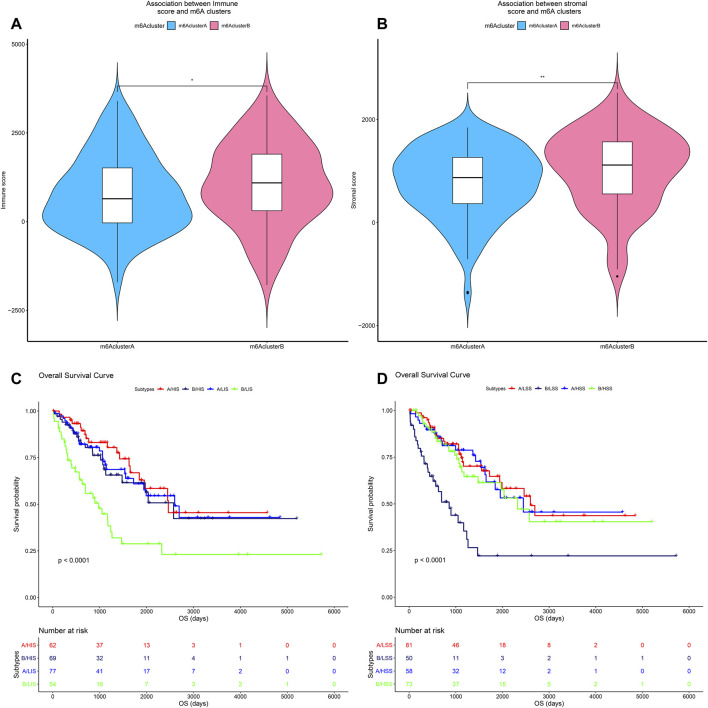
Effects of different m6A modification patterns on the immune infiltration of STS. **(A)** Comparison of immune score among m6A clusters A and B; **(B)** Comparison of stromal score among m6A clusters A and B; **(C)** survival analysis of different immune scores among m6A clusters A and B (A: m6A cluster A, B: m6A cluster B, LSS, low immune score, and HSS, high immune score); and **(D)** survival analysis of different stromal scores among m6A clusters A and B. (A: m6A cluster A, B: m6A cluster B, LIS: low stromal score, and HIS, high stromal score); *p* <0.05* and *p* <0.01**.

In order to understand the influence of m6A modification patterns on the STS microenvironment, the immune cell infiltration in TCGA-SARC is shown in Figure 5A. The correlation plot of each immune cell is also displayed in Figure 5B. CD8 T cells and follicular helper T cells had higher correlation with other cells. The relationships between 22 kinds of immune cells and different m6A modification patterns were analyzed by the Kruskal–Wallis test, respectively (Figure 5C). m6A cluster A showed higher infiltration of M1 macrophage, CD8 T cell, and NK cell, while the M2 macrophage showed higher infiltration in m6A cluster B. M2 macrophage (*p* < 0.050), mast cell activated (*p* < 0.050), mast cell resting (*p* < 0.0001), neutrophils (*p* < 0.050), and T cell CD4 memory activated (*p* < 0.010) were significantly correlated with different m6A modification patterns in STS. In addition, the immune and stromal scores of m6A cluster A/B were separately calculated, and the results are shown in Figures 6A,B. Compared with m6A cluster A, m6A cluster B was characterized by a significantly higher immune score (*p* < 0.050) and stromal score (*p* < 0.010). Survival analysis of different m6A modification patterns in different immune scores and stromal scores was subsequently performed, and the results are shown in Figures 6C,D. Significant prognostic differences were found in different m6A modification patterns and immune/stromal score (*p* < 0.0001). The Cox regression analysis also showed that different m6A modification patterns were significantly correlated with the prognosis of STS in different immune scores (*p* = 0.001) and stromal scores (*p* < 0.001), respectively. In addition, differences in the microenvironment between m6A cluster A/B and normal adjacent tissue were also explored in GSE17674. As shown in Supplementary Figure S4A, consensus clustering analysis also identified two distinct m6A modification patterns. The immune score of both m6A clusters A and B was higher than that of normal tissue with *p* = 0.159 and *p* = 0.034, respectively (Supplementary Figures S4B,C). The aforementioned results suggested that different m6A modification patterns affected the immune infiltration in the STS microenvironment, which further affected the prognosis of STS.

### Construction of m6A Score

**FIGURE 7:**
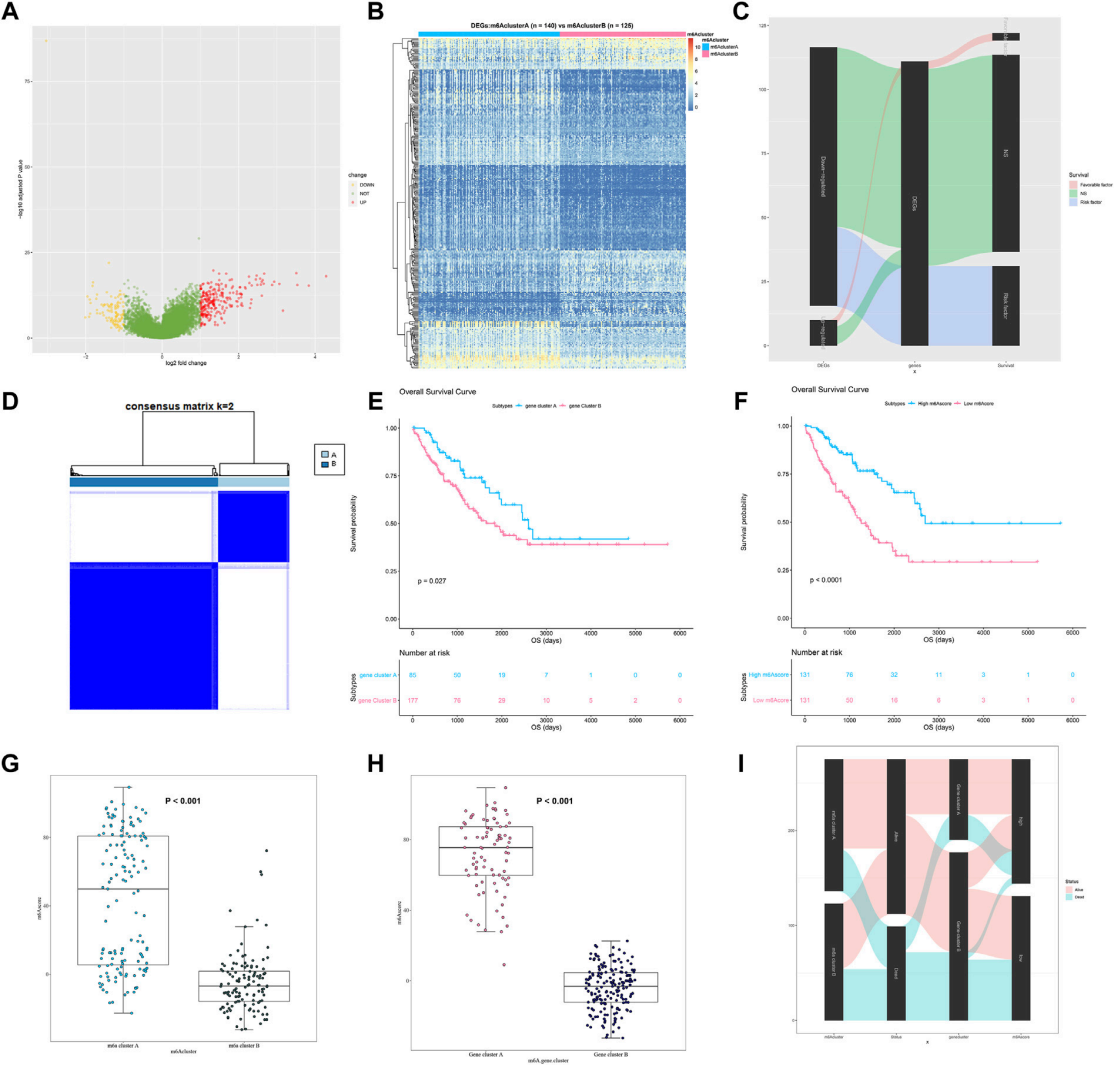
Construction of m6Ascore. **(A)** Volcano plot of DEGs between m6A cluster A and B; **(B)** the heatmap of the expression of DEGs in m6A cluster A and B; **(C)** the relationship between DEGs and these prognostic genes visualized as a Sankey diagram; **(D)** the result of consensus clustering analysis in STS based on 328 DEGs; **(E)** survival plot of gene clusters A and B in TCGA-SARC (*p* = 0.027); **(F)** survival plot of high and low m6A score in TCGA-SARC (*p* < 0.0001); **(G)** comparison of m6A score among m6A clusters A and B; **(H)** comparison of m6Ascore among gene clusters A and B; and **(I)** the relationship between m6A clusters, gene cluster survival status, and m6A score visualized as a Sankey diagram.

A total of 328 DEGs between different m6A modification patterns were determined. The full list of 328 DEGs is shown in Supplementary Table S5. Among them, 227 genes were upregulated in m6A cluster A while 101 genes were upregulated in m6A cluster B (Figure 7A). The GO and KEGG enrichment analysis was also performed, and the results are shown in Supplementary Tables S6, S7. Similar to the result of m6A regulators, these DEGs were enriched in the p53 signaling pathway (*p* = 0.002), ECM–receptor interaction (*p* = 0.005), and PI3K-Akt signaling pathway (*p* = 0.023), which were closely related to the progression of STS. The heatmap of these DEGs in STS is also shown in Figure 7B. Cmap was analyzed according to these 328 DEGs, and the results are shown in Supplementary Table S8. Imatinib (*p* = 0.001) and furazolidone (*p* = 0.025) were regarded as important targets treating STS. Univariate Cox regression analysis was further performed to identify prognostic genes among these 328 DEGs. Of the 328 DEGs, 90 of them were identified as prognostic genes (*p* < 0.050). Furthermore, 58 genes were identified as protective genes due to HR < 1, while 32 genes were regarded as risk genes based on HR > 1. The full list of prognostic DEGs is displayed in Supplementary Table S9. The relationships between DEGs and these prognostic genes are visualized in a Sankey diagram in Figure 7C. To our surprise, all protective DEGs, upregulated in m6A cluster A, were from m6A cluster A. Contrary to m6A cluster A, all risk DEGs, upregulated in m6A cluster B, belonged to m6A cluster B. This was also consistent with the result of the survival analysis. To further evaluate the stability of m6A modification patterns, consensus cluster analysis was performed based on 328 DEGs, and two distinct gene clusters A and B were identified (Figure 7D). The corresponding cumulative distribution function plot and delta area plot for clustering are also shown in Supplementary Figures S5A, B. Among them, 92.9% samples of gene cluster A were from m6A cluster A while 66.7% samples of gene cluster B were from m6A cluster B, which also implied that the identification of m6A modification patterns was relatively stable. The survival plot between different gene clusters is also shown in Figure 7E. Gene cluster A had a better prognosis of STS than gene cluster B (*p* = 0.027), which was also consistent with the survival result of m6A clusters. The Cox regression analysis indicated that different gene clusters were significantly correlated with the prognosis of STS (*p* = 0.029). The 5-year survival rates of gene clusters A and B were 66.0 and 50.0%, respectively. Due to the significant difference in immune infiltration and prognosis between m6A modification patterns, m6A score was constructed to quantify the modification pattern of each STS sample. Then, STS samples were divided into high- and low-m6A score groups. Similar to what we found in gene clusters, 75.7% samples of high m6A score were from m6A cluster A while 78.4% samples of low m6A score were from m6A cluster B. The survival plot between high and low m6A score is also shown in Figure 7F. A high m6A score had a better prognosis of STS than a low m6A score (*p* < 0.0001). The Cox regression analysis indicated that different m6A scores were significantly correlated with the prognosis of STS (*p* < 0.001). The m6A score was also compared in different m6A clusters and gene clusters, and the result is shown in Figures 7G,H. The high-m6A score group showed a significantly better outcome than the low-m6A score group (*p* < 0.0001), and the result was the same in gene cluster A (*p* < 0.001). In addition, in Supplementary Figure S6, the area under the curve (AUC) of m6A score for prediction of the 1-, 3-, and 5-year survival of STS was 0.77, 0.71, and 0.68, respectively. Finally, a Sankey diagram was performed to summarize the correlation between m6A clusters, gene clusters, m6A score, and prognosis of STS (Figure 7I). It could be clearly seen that m6A cluster A, gene cluster A, and high m6A score were correlated with a better prognosis of STS while m6A cluster B, gene cluster B, and low m6A score were related to a poorer prognosis of STS. The aforementioned results indicated that the m6A score could accurately distinguish the m6A modification patterns and predict the prognosis of STS.

### m6A score Could Predict Immunotherapy Response

**FIGURE 8:**
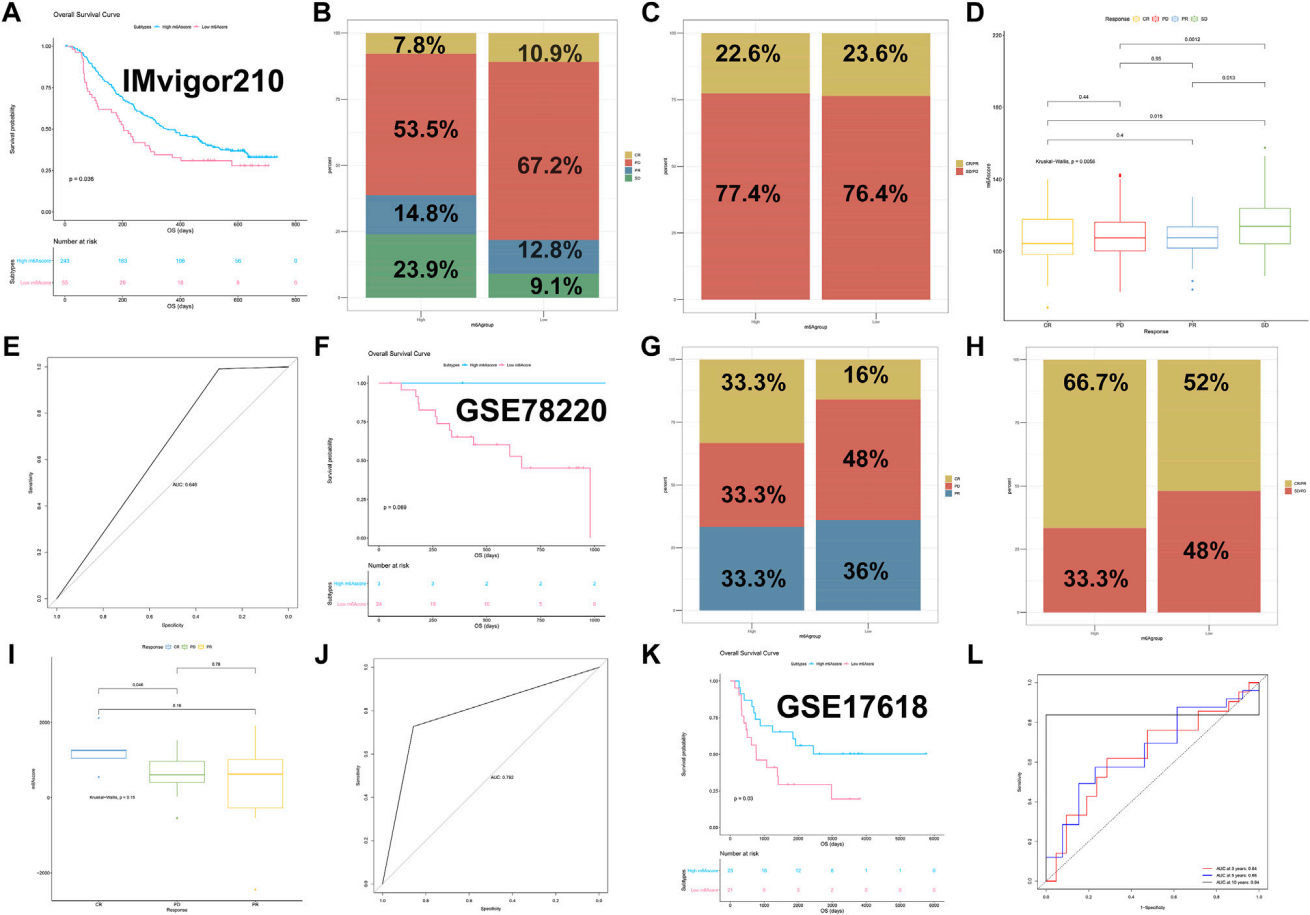
The m6Ascore predicted immune response in immunotherapy datasets and validation of m6Ascore. **(A)** Survival plot of high and low m6A score in the IMvigor210 dataset (*p* = 0.036); **(B)** relative percent of immune responses in high and low m6A score in the IMvigor210 dataset; CR, complete response; PR, partial response; SD, stable disease; PD, progressive disease; **(C)** relative percent of CR/PR and SD/PD in high and low m6A score in the IMvigor210 dataset; CR, complete response, PR, partial response, SD, stable disease, PD, progressive disease; **(D)** box plots of m6Ascore in different immune responses in the IMvigor210 dataset; **(E)** ROC curve for prediction of immune response in the IMvigor210 dataset; **(F)** survival plot of high and low m6A score in the GSE78220 dataset (*p* = 0.069); **(G)** relative percent of immune responses in high and low m6A score in the GSE78220 dataset; CR, complete response; PR, partial response; PD, progressive disease; **(H)** relative percent of CR/PR and SD/PD in high and low m6A score in the GSE78220 dataset; CR, complete response; PR, partial response; PD, progressive disease; **(I)** box plots of m6A score in different immune responses in the GSE78220 dataset; **(J)** ROC curve for prediction of immune response in the GSE78220 dataset; **(K)** survival plot of high and low m6A score in the GSE17618 dataset (*p* = 0.030); and **(L)** ROC curve for prediction of the 3-, 5-, and 10-year survival of STS in the GSE17618 dataset.

Considering that m6A score could well predict the prognosis of STS, we further explore whether m6A score could effectively predict the response to ICB in IMvigor210 and GSE78220 datasets. For the IMvigor210 dataset, 298 samples were divided into high-m6A score (*n* = 243) and low-m6A score groups (*n* = 55). The result of the survival analysis turned out that a high m6A score showed a better prognosis than a low m6A score (Figure 8A, *p* = 0.036). The corresponding Cox regression analysis also revealed that different m6A scores were significantly correlated with the prognosis of each sample in IMvigor210 (*p* = 0.037). Furthermore, the relative percent of complete response (CR), partial response (PR), progression disease (PD), and stable disease (SD) in high- and low-m6A score groups were compared, and the results are shown in Figures 8B–D. 53.4% of the high-m6A score group had PD, while 67.2% of the low-m6A score group had PD, and patients with SD had higher m6A score than patients with PD (*p* = 0.001). The ROC analysis for the prediction of the response to anti-PD-L1 is shown in Figure 8E, and the AUC was 0.646. For the GSE78220 dataset, 27 samples were divided into high-m6A score (*n* = 3) and low-m6A score groups (*n* = 24). Survival analysis was further performed between different m6A score groups. Similar to IMvigor210, the high-m6A score group showed a better prognosis than the low-m6A score group (*p* = 0.069, Figure 8F). The corresponding Cox regression analysis also revealed that different m6A scores were associated with the prognosis of each sample in GSE78220 (*p* = 0.018). The relative percent of CR, PR, and PD in the high-m6A score group were all 33.3%, while in the low-m6Ascore group, the proportion was 16.0, 48.0,, and 36%, respectively (Figures 8G,H). The m6A score did not show a significant difference among samples with different immunotherapy responses (Figure 8I), which could be due to the small sample size of GSE78220. The ROC curve for prediction of the response to anti-PD-1 is shown in Figure 8J, and the AUC was 0.792.In addition, the independent sarcoma dataset GSE17618 was used for the validation of the m6Ascore. GSE171618 dataset was divided into high- (*n* = 23) and low-m6A score groups (*n* = 21). As in Figure 8K, the high-m6A score group showed a better prognosis than the low-m6A score group (*p* = 0.030). The corresponding Cox regression analysis also indicated that different m6A scores were significantly correlated with the prognosis of STS in GSE17618 (*p* = 0.034). The 5-year survival rates of the high- and low-m6A score groups were 55.9 and 29.3%, respectively. The AUC for prediction of the 3-, 5-, and 10-year survival of STS was 0.64, 0.66, and 0.84, respectively (Figure 8L). The event-free survival curve between high and low m6A score is also displayed in Supplementary Figure S7. Similar to the result of overall survival, the high-m6A score group had a better prognosis (*p* = 0.074). The corresponding Cox regression analysis also indicated that different m6A scores were significantly correlated with the event-free survival of STS in GSE17618 (*p* = 0.070). In general, our study revealed a non-negligible role of m6A modification in the STS microenvironment, which could well predict the response to PD-1/PD-L1 immunotherapy.

## Discussion

Due to diverse histopathological classification, STS has not been fully understood so far, which received widespread attention (Trojani et al., 1984). A previous study constructed an m6A-related risk model to predict the prognosis of STS (Hou et al., 2020). However, different m6A modification patterns were not analyzed comprehensively. Recent studies also revealed the significant role of m6A modification in gastric cancer and pancreatic cancer (Zhou et al., 2021). However, systematic analysis of the m6A modification in STS was still rare.

The CNV analysis indicated that ZC3H13 and ELAVL1 had the highest variant frequency. A recent study also indicated that ZC3H13 could inhibit colorectal cancer through Ras-ERK pathways (Zhu et al., 2019). Moreover, ELVAL1 knockout mice were also found to have lower tumor growth in a previous report (Chang et al., 2014). Therefore, our findings suggested that these regulators might also play a key role in STS progression. Besides, 96% of m6A regulators were found to have significantly different expressions between STS and normal samples, which was consistent with a former study that m6A regulators expressed differently in colorectal cancer based on bioinformatics analysis (Zhang et al., 2021a). These results also suggested that m6A regulation might exist in various types of cancer. Univariate Cox regression analysis showed that IGF2BP1 and YTHDF2 were top significantly correlated with the poor prognosis of STS. Previous studies also found that IGFBP2 could accelerate the migration of tumor cells by regulating LEF1 and SNAI2 (Zirkel et al., 2013). Moreover, IGF2BP1 was also regarded as a risk factor in neuroblastoma (Bell et al., 2015). YTHDF2 was also related to the poor prognosis of glioma in Lin et al.’s (2020) research, and the latest research implied that stabilized YTHDF2 could enhance the growth capacity of glioblastoma (Fang et al., 2021). These studies were consistent with our results, which also revealed the potential prognostic value of IGFBP1 and YTHDF2 in STS.

Furthermore, two m6A modification patterns in STS were identified. m6A cluster A was correlated with DNA replication, mismatch repair, and a better prognosis, while m6A cluster B was related to MYC, EMT signaling pathways, and a poor prognosis. As an important part of the cell cycle, stable DNA replication was key to the normal activities of cells. Dysfunction of DNA replication might result in the occurrence of diseases. For example, abnormal replication of MCM10 would lead to NK cell deficiency (Mace et al., 2020). A recent study also reported that DNA replication stress could be used to enhance the antitumor ability in squamous cell carcinoma (Zhang et al., 2021b). In addition, mismatch repair (MMR) was also known for its tumor suppressor function. For instance, dysfunction of MMR genes would lead to Lynch syndrome, which was susceptible to cancer including ovarian cancer (Zhang et al., 2021b). MMR deficiency was also related to the occurrence of endometrial cancer (McDougal et al., 2021). The MYC gene was a proto-oncogene, which has been studied for many decades (Cole and McMahon, 1999). A recent study showed that MYC could be regulated by USP16, which further inhibited prostate cancer progression (Ge et al., 2021). MYC was also found to participate in angiogenesis (Meškytė et al., 2020). EMT was considered to be related to tumor metastasis. A previous study reported that EMT induced by HOXA10 contributed to gastric cancer metastasis (Song and Zhou, 2021). These studies supported our results, which provided novel insights into the role of m6A modification in STS. The immune cell infiltration analysis showed that M1 macrophage, CD8 T cell, and NK cell were enriched in m6A cluster A while M2 macrophage was enriched in m6A cluster B. M1 macrophages were generally thought to inhibit tumor growth while M2 macrophages were found to promote tumor progression, which was also proved in many studies. For example, M1 macrophage could inhibit colon cancer growth (Engström et al., 2014). In Jackute et al.’s (2018) study, low infiltration of M2 macrophages was correlated with an improved prognosis of lung cancer. Improved CD8 T cell infiltration was also considered to be correlated with a better prognosis (Li et al., 2021b). As a part of innate immunity, NK cells also played an anti-tumor role. Recent research reported that NK cells could inhibit lung tumor growth in mice models (Yamamoto et al., 2018).In addition, both m6A clusters A/B showed higher immune scores than normal tissue. This may be due to the local inflammatory and immune response of tumor tissue, which recruits more immune cells, while there is no tumor-mediated immune cell aggregation in normal tissue. For example, a recent study revealed that macrophages could be recruited for breast cancer by increasing CCL2 (Wolfsberger et al., 2021). A former study also indicated that many neutrophils were recruited in non-small-cell lung cancer (Mollaoglu et al., 2018). Our findings were consistent with these studies, indicating that the m6A cluster A was related to a strong anti-tumor immune response while m6A cluster B was related to a suppressed anti-tumor immune response.

The result of Cmap analysis showed imatinib and furazolidone as important drugs for treating STS. A recent study illustrated that furazolidone could induce apoptosis in lung cancer by downregulating NF-kappa B (Yu et al., 2020). Furazolidone was also found to prevent the growth of hepatoma cells by enhancing reactive oxygen species (Sun et al., 2015). Furazolidone was also found to be a potential drug for the treatment of acute myeloid leukemia by enhancing the expression of p53 (Jiang et al., 2013). Imatinib was found to improve the prognosis of gastrointestinal stromal tumors (Kurtovic-Kozaric et al., 2017). A recent study also implied that imatinib might slow down the progression of leukemia (Druker et al., 2001). Given the important role these drugs played in other tumors, they might also become promising drugs for STS treatment. We further identified two gene clusters based on DEGs between m6A clusters A and B. Similar to our m6A cluster modification patterns, gene cluster A showed a better prognosis of STS, which also validated that our previous m6A modification patterns were reliable. The m6Ascore was significantly higher in m6A cluster A and gene cluster A, respectively. Moreover, the AUC of m6Ascore for predicting the 1-, 3-, and 5-year survival of STS was 0.77, 0.71, and 0.68. In addition, the survival results of validation dataset GSE17618 also showed that a high m6Ascore was related to a better prognosis, and the AUC for predicting the 3-, 5-, and 10-year survival was 0.64, 0.66, and 0.84, respectively. These results demonstrated that the m6A score could identify different m6A modification patterns and be used as a potential prognostic indicator in STS.

Our results also showed that m6A score could predict the response to immunotherapy. A high m6A score was correlated with a better immunotherapy response. Previous studies focused on suppressing macrophages to obtain a better immunotherapy response (Dong et al., 2021) and adding nanoparticles into the TME to further strengthen the anti-tumor ability (Yang et al., 2021). These studies mainly changed the immunotherapy response by affecting the components of the TME. However, biomarkers to directly predict the response of immunotherapy were still rare. Here, we reported the AUC of m6A score for prediction of immune response to PD-1/L1 was 0.646 and 0.794, respectively. Therefore, our m6A score could also be a promising predictor for tumor immunotherapy. In addition, the m6A score was also validated by an independent sarcoma dataset, which also proved that the m6A score was reliable.

Our research also had some limitations. More immunotherapy datasets were needed to validate the m6A score. In addition, more clinical trials were also needed to further validate the drugs and cancer-related pathways we screened in this study.

## Conclusion

In general, the m6A modification patterns in the STS microenvironment were comprehensively analyzed. An m6A score to evaluate different m6A modification patterns was established through integrative analysis. A high m6A score showed a better prognosis of STS, while a low m6Ascore led to a poor prognosis of STS. In addition, the m6A score was validated by an independent dataset successfully and accurately predicted the prognosis of STS, which could be a promising predictor for cancer immunotherapy.

## Data Availability

The original contributions presented in this study are included in the article/Supplementary Material, further inquiries can be directed to the corresponding author.

## Supplementary Material

Supplementary Figure S1 The flowchart of this study.

Supplementary Figure S2 The expression of 25 m6A regulators between HSMC and A673 cells; (A–Y) the expression of different m6A regulators between HSMC and A673 cells. p < 0.05*, p < 0.01**, p < 0.001***, and p = 0****.

Supplementary Figure S3 The cumulative distribution function plot and delta area plot for clustering in TCGA-SARC. **(A)** The cumulative distribution function plot in TCGA-SARC; **(B)** the delta area plot for clustering in TCGA-SARC.

Supplementary Figure S4 Exploration of differences in the microenvironment between different m6A modification patterns and normal tissue in GSE17674. **(A)** The result of consensus clustering analysis in GSE17674; **(B)** comparison of immune score between m6A cluster A and normal tissue in GSE17674; and **(C)** comparison of immune score between m6A cluster B and normal tissue in GSE17674.

Supplementary Figure S5 The cumulative distribution function plot and delta area plot for clustering in TCGA-SARC based on 328 DEGs. **(A)** The cumulative distribution function plot in TCGA-SARC based on 328 DEGs; **(B)** the delta area plot for clustering in TCGA-SARC based on 328 DEGs.

Supplementary Figure S6 The ROC curve of m6A score for prediction of the 1-, 3-, and 5-years survival of STS in TCGA-SARC.

Supplementary Figure S7 The event-free survival plot of high and low m6A score in the GSE17618 dataset (p = 0.074)

## Abbreviations

AUC, area under the curve; cMap, Connectivity Map; CNV, copy number variation; DEGs, differentially expressed genes; ES, enrichment score; FDR, false discovery rate; FPKM, Fragments Per Kilobase Million; GEO, Gene Expression Omnibus, GO, Gene Ontology; GSEA, Gene Set Enrichment Analysis; HR, hazard ratio; HSMC, human skeletal muscle cell; KEGG, Kyoto Encyclopedia of Genes and Genomes; m6A, N6-methyladenosine; PCA, principal component analysis; PPI, protein–protein interaction; ROC, receiver operating characteristic; RT-PCR, real-time polymerase chain reaction; STRING, Search Tool for Recurring Instances of Neighbouring Genes; STS, soft tissue sarcoma; TCGA, The Cancer Genome Atlas; TME, tumor microenvironment.
