# Supplementary figures and images for: Molecular Characteristics of m6A Regulators and Tumor Microenvironment Infiltration in Soft Tissue Sarcoma: A Gene-Based Study
Source: Front Bioeng Biotechnol. 2022 Apr 19;10:846812. doi: 10.3389/fbioe.2022.846812 (PMC9062003; doi:10.3389/fbioe.2022.846812)

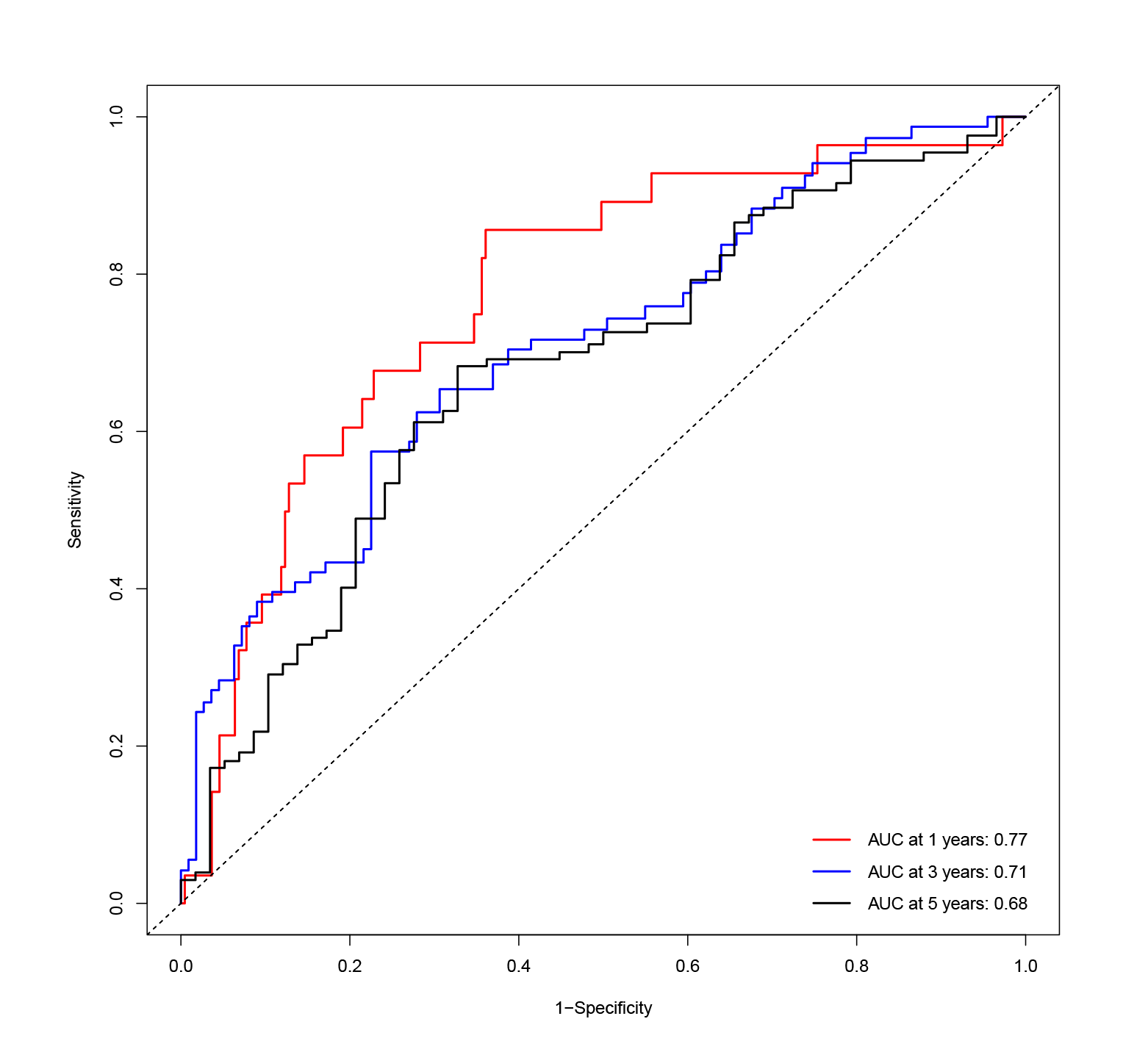

Supplement: Supplementary file 1 [file Image6.TIF]

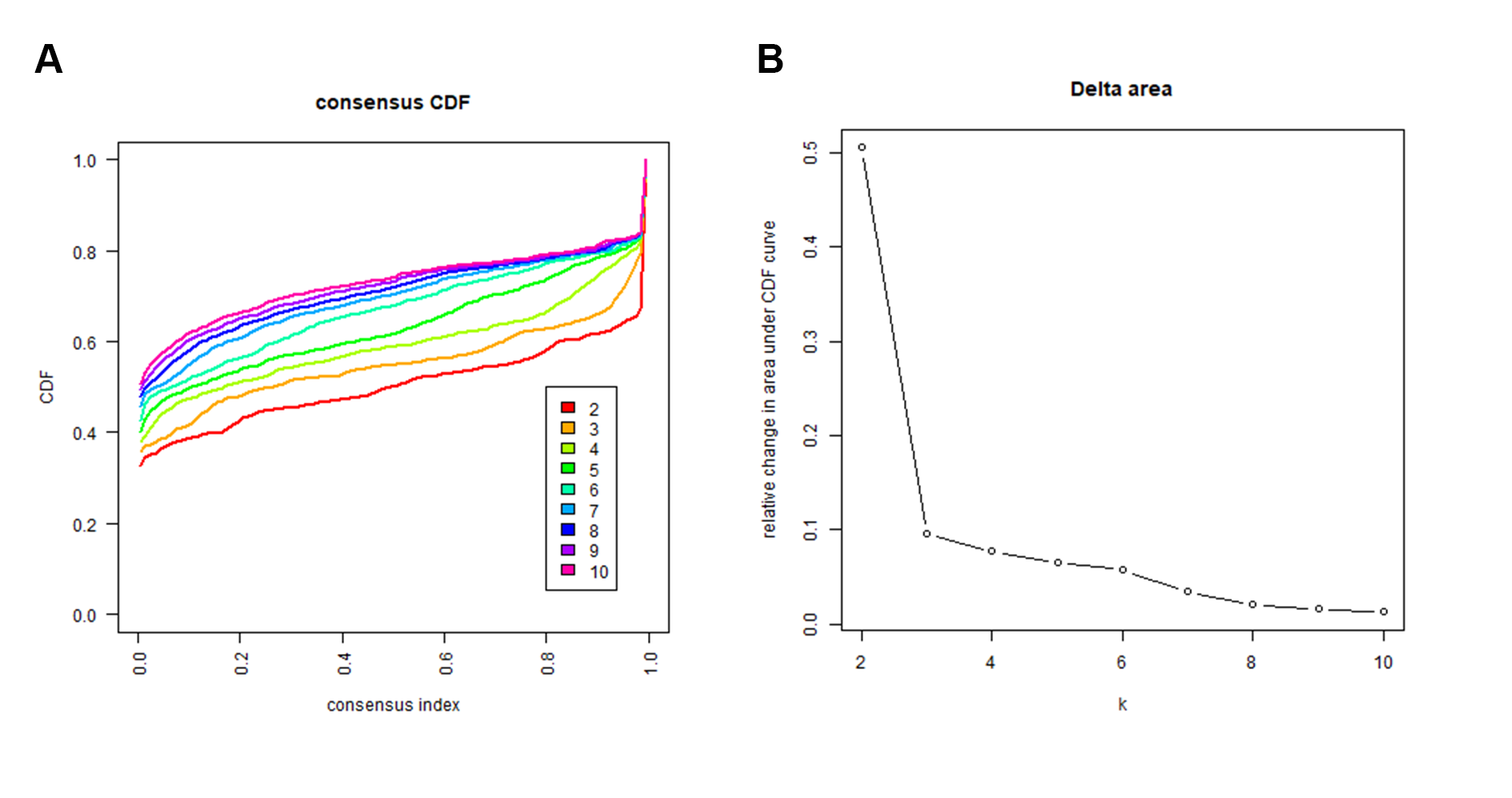

Supplement: Supplementary file 2 [file Image3.TIF]

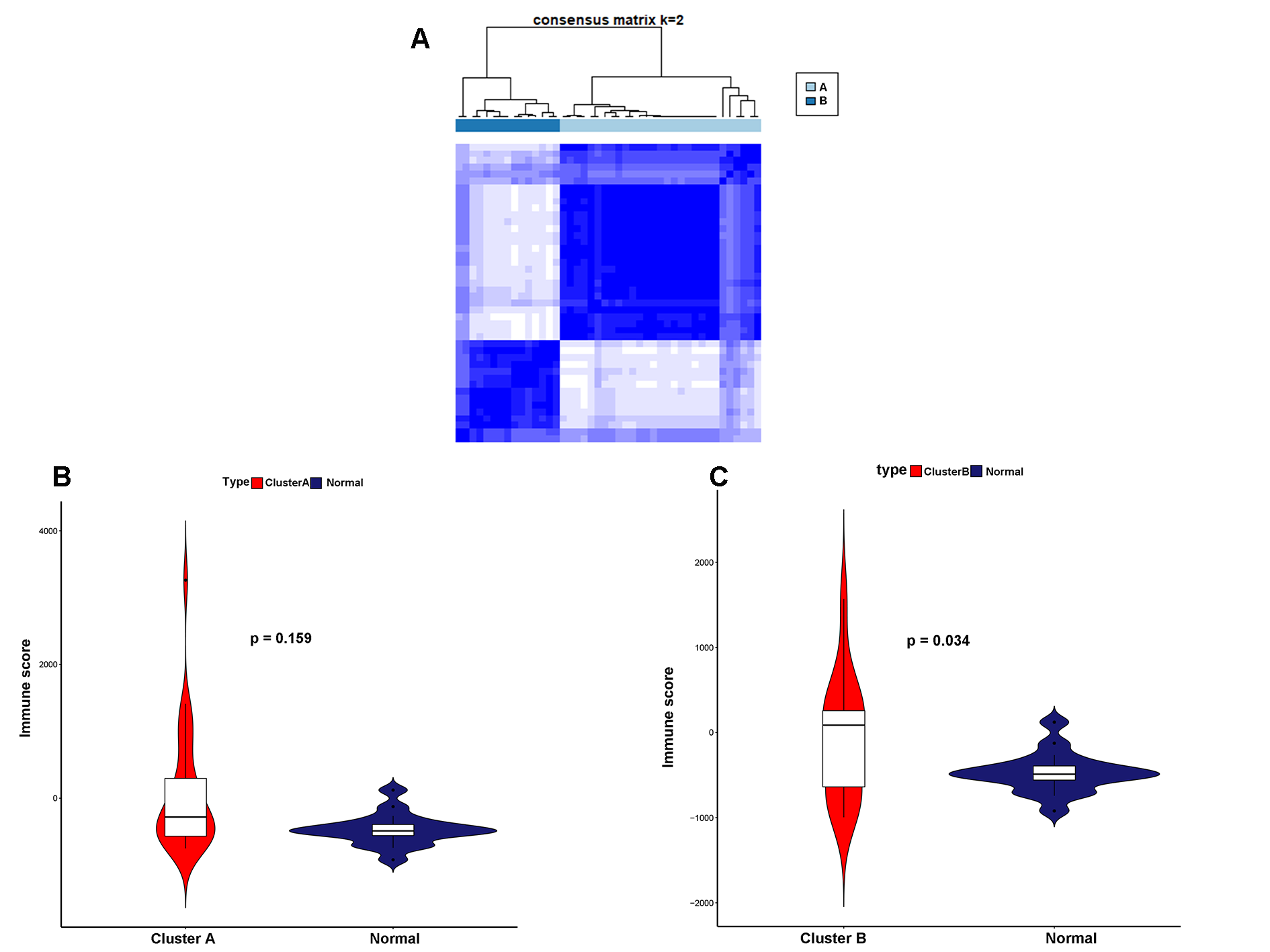

Supplement: Supplementary file 3 [file Image4.TIF]

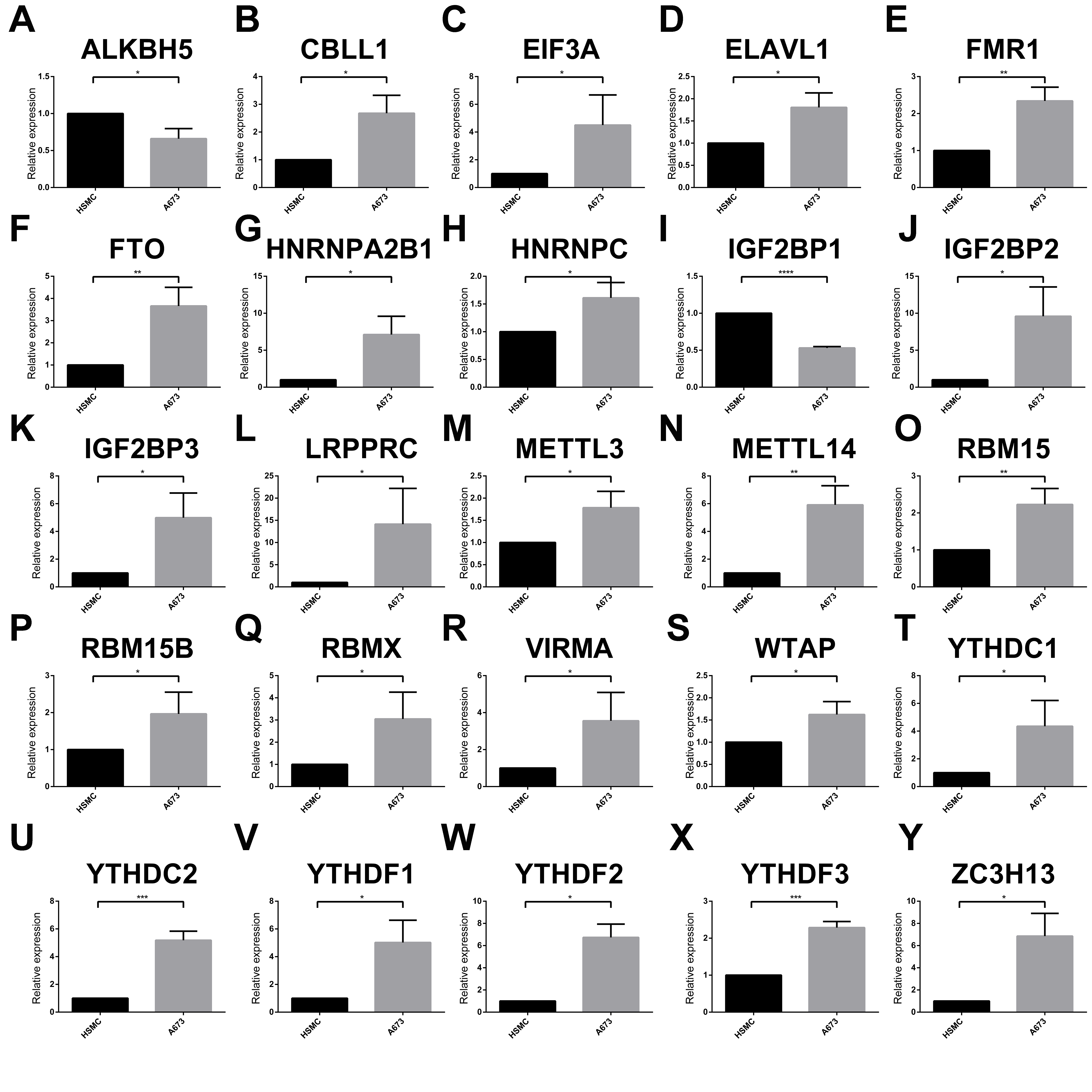

Supplement: Supplementary file 4 [file Image2.TIF]

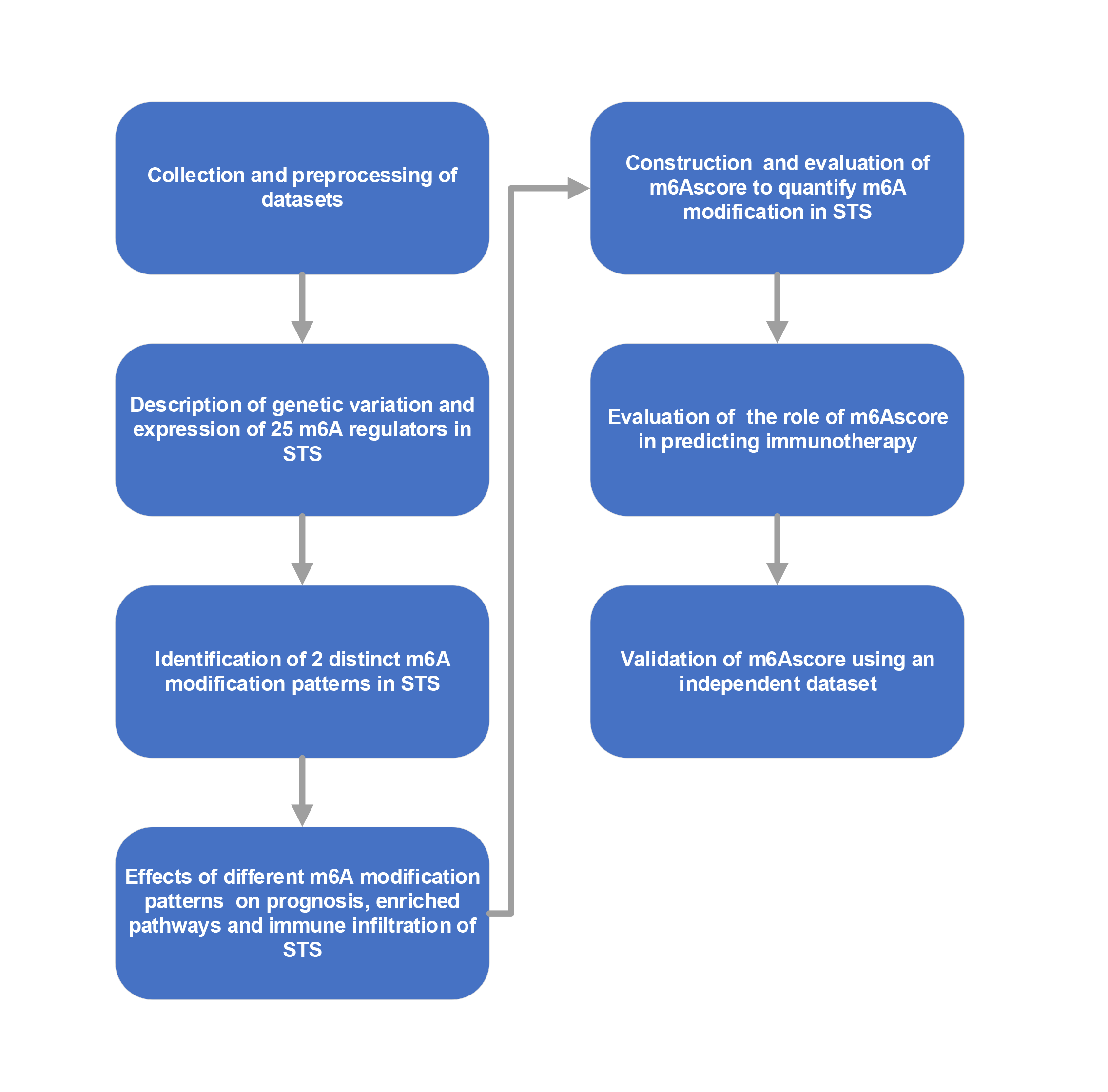

Supplement: Supplementary file 5 [file Image1.TIF]

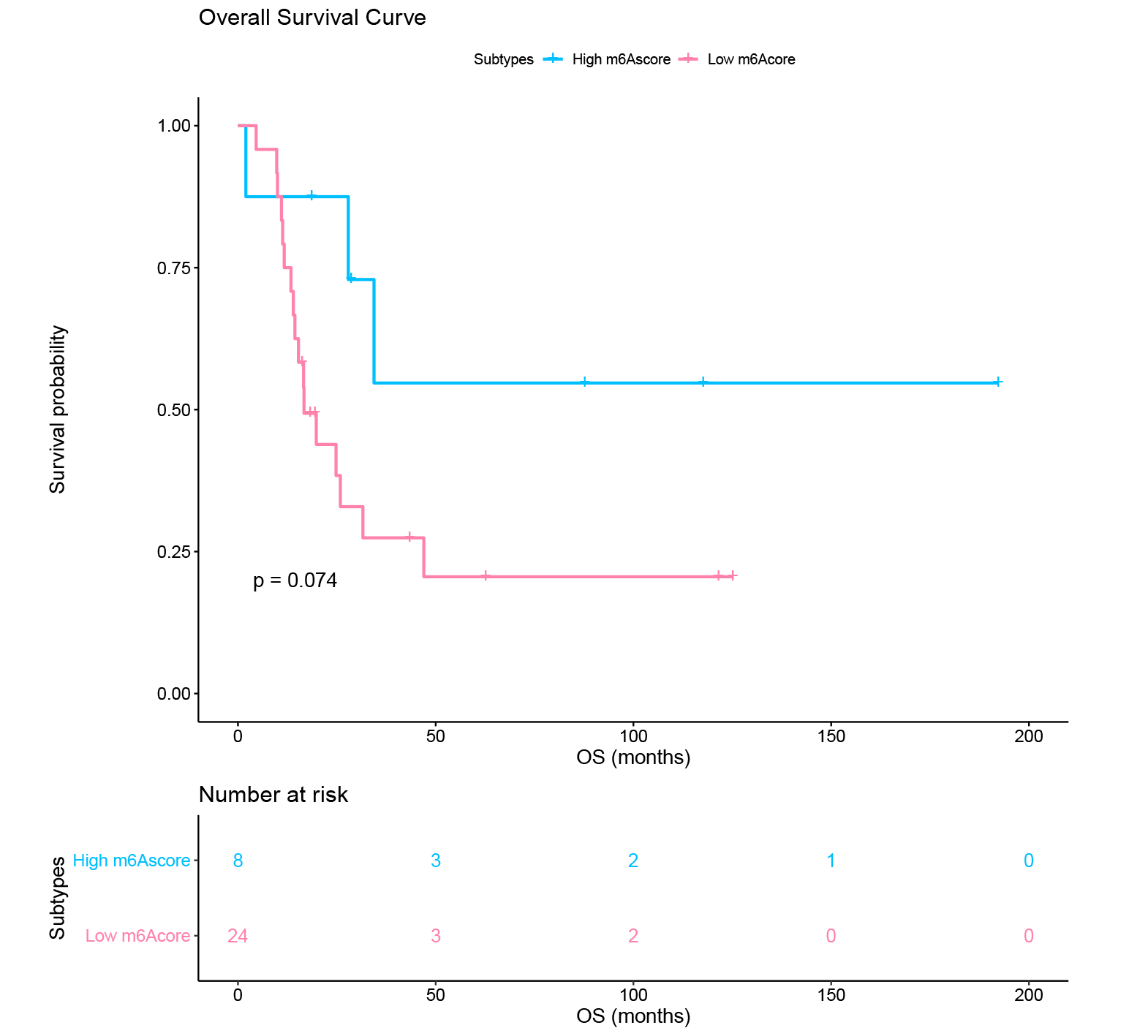

Supplement: Supplementary file 6 [file Image7.TIF]

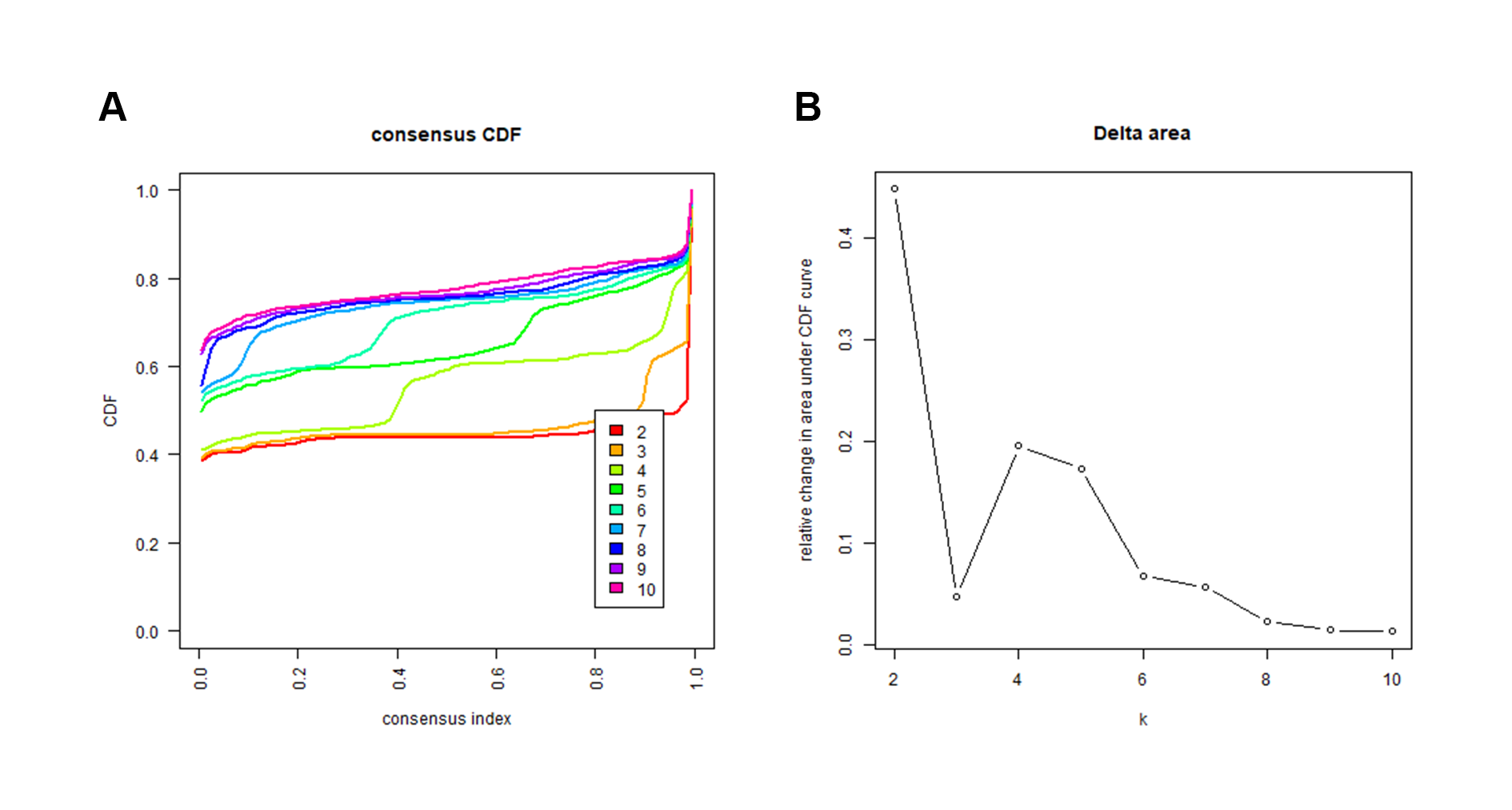

Supplement: Supplementary file 8 [file Image5.TIF]
